# Phase-locking, quasiperiodicity and chaos in periodically driven noisy neuronal models: a spectral approach

**DOI:** 10.1186/1471-2202-13-S1-P64

**Published:** 2012-07-16

**Authors:** Alla Borisyuk, Firas Rassoul-Agha

**Affiliations:** 1 Department of Mathematics, University of Utah, Salt Lake City, UT 84112, USA

## 

In auditory experiments neurons are often driven by periodic or periodically modulated inputs. The response of the neurons is often periodic and phase-locked to the stimulus. However, this is not always the case. It is well-known that even simple deterministic models can exhibit quasiperiodic behavior, with no clear relationship between the phases of the stimulus and response. Moreover, the biological situation is further complicated by presence of noise in the periodic inputs and intrinsic cellular properties, e.g. firing rate adaptation. We develop analytical tools for detection of parameter regimes corresponding to different locking behaviors in more detailed stochastic biophysical models, so that these regimes can be avoided or chosen as necessary.

In an effort to develop a mathematical theory applicable to the above biologically-motivated project on phase-locking and related phenomena, we have developed a spectral approach to stochastic circle maps. A stochastic circle map is defined as a Markov chain on the circle. This abstract class of objects includes a wide range of models for firing times of periodically forced noisy neuronal models.

We define and analyze some dynamic properties of the system such as stochastic phase locking and stochastic bifurcation cascades. The main tool is analysis of the spectrum and eigenspaces of the transition operator of the Markov chain. We relate the dynamics of the stochastic system to those of a few identifiable finite state deterministic dynamical systems, the dynamics of which can in turn be deduced from the spectral properties of the corresponding matrices. We also show that the firing regime of the stochastic system can be determined by the shape of its eigenvalue “cloud.”

